# Supplementary material for: Factors contributing to differences in physical activity levels in (pre)frail older adults living in rural areas of China
Source: PLoS One. 2025 Nov 4;20(11):e0335607. doi: 10.1371/journal.pone.0335607 (PMC12585090; doi:10.1371/journal.pone.0335607)
Supplement: S1 File — (DOCX) [file pone.0335607.s001.docx]

**Supplementary Material - Appendixes**

Table S1: Measurement Instruments and variables

| Variables (unit) | Measurement | Meaning |
| --- | --- | --- |
| Age | records | >= 60 years old |
| Sex | records | 1=female; 0=male |
| Occupation | records | 1=Farmer; 0=Worker/Individual Business/Driver/Retirement/Unemployed |
| Education (year) | records | 1= <=6y; 0 = >6y |
| Income (￥/m) | records | 1=<3000; 0=>=3000 |
| Marriage | records | 1=Married; 0=Single/Divorced;/Widow |
| Living | records | 1=Alone; 0=Cohabitation/With Children/Others |
| Smoking | self-reported | 1=yes; 0=no |
| Drinking | self-reported | 1=yes; 0=no |
| Daily fiber | self-reported | 1=yes; 0=no |
| Daily protein | self-reported | 1=yes; 0=no |
| Comorbidity | self-reported | 1=>=5 disease; 0=<5 diseases |
| Ambulation | self-reported | 1=could walk 500 meter;  0= could not walk 500 meter |
| Functional strength | self-reported | Could go up a one floor stair or a 10-meter slope  1= yes  0=no |
| General Health | self-reported | 1=Bad;  0=Well/Good/Great/Excellent |
| Cognitive impairment (CI) | physical test | 0=no; 1=yes; defined by total score of MMSE and education level; <18 for illiterate; <21 for 1-6 years of education; <25 for 7 or more years of education [39] |
| WHR | physical test | Calculated by waistline (cm)/hipline (cm) |
| BMI | physical test | Calculated by weight/(height/100)2 |
| GP (kg) | physical test | Maximum value of two measurements in each hand |
| WS (m/s) | physical test | Walking speed, measured by 3m walking test |
| TUG (seconds) | physical test | Time of Time Up and Go test – total time |
| SPPB - | physical test | Total score of the Short Physical Performance Battery. Range 0-12  12 points: Highest performance, indicating optima lower extremity function and mobility.  0-3 points: Very low performance, indicating severe functional impairments and a high likelihood of disability, falls, or dependency. |
| MMSE | cognitive test | Total score of mini-mental state examination, range 0-30  See CI |
| PHQ-9 | self-reported | Total score of Patient Health Questionnaire-9, range 0-27 –  0–4: Minimal or no depression;  5–9: Mild depression;  10–14: Moderate depression;  15–19: Moderately severe depression;  20–27: Severe depression |
| GAD-7 | self-reported | Total score of General Anxiety Disorder Questionnaire-7, range 0-21;  0–4: Minimal anxiety  5–9: Mild anxiety  10–14: Moderate anxiety  15–21: Severe anxiety |
| SSRS | self-reported | Total score of Social Support Rate Scale questionnaire score, range 0-66 - The total score for each domain can range from 0 to 100,  Low Score – Indicates weak social support and possible isolation.  Moderate Score – Suggests a moderate level of perceived social support.  High Score – Reflects strong social support, which is beneficial for emotional and psychological well-being. |
| IPAQ-SF | self-reported | Physical activity level calculated by Short Form International Physical Activity  1=low;  2=moderate;  3=high. |

Table S2: Hyperparameters settings for the XGBoost model

| Parameter | Tuning range | Best parameter |
| --- | --- | --- |
| Eta | 0.005, 0.01, 0.05, 0.1 | 0.01 |
| Max depth | 2, 4, 6, 8 | 8 |
| Nrounds | 50,100, 200 | 200 |
| Gamma | 0.005, 0.1, 1, 3 | 0.005 |
| Colsample_bytree | 0.3, 0.5, 0.7, 0.9 | 0.7 |
| Min child weight | 1, 3, 5, 7 | 1 |
| SubSample | 0.3, 0.5, 0.7, 0.9 | 0.7 |
