# Supplementary material for: Factors contributing to differences in physical activity levels in (pre)frail older adults living in rural areas of China
Source: PLoS One. 2025 Nov 4;20(11):e0335607. doi: 10.1371/journal.pone.0335607 (PMC12585090; doi:10.1371/journal.pone.0335607)
Supplement: Attachment — Submitted filename: Response to Reviewers.docx [file pone.0335607.s004.docx]

PONE-D-25-34585

Factors contributing to differences in physical activity levels in (pre)frail older adults living in rural areas of China

PLOS ONE

Editor’s Comments

Please include the following items when submitting your revised manuscript:

**A rebuttal letter** that responds to each point raised by the academic editor and reviewer(s). You should upload this letter as a separate file **labeled 'Response to Reviewers'**.

**A marked-up copy** of your manuscript that highlights changes made to the original version. You should upload this as a separate file **labeled 'Revised Manuscript with Track Changes'**.

**An unmarked version** of your revised paper without tracked changes. You should upload this as a separate file labeled '**Manuscript**'.

If you would like to make changes to your financial disclosure, please include your updated statement in your cover letter. Guidelines for resubmitting your figure files are available below the reviewer comments at the end of this letter.

1. Please ensure that your manuscript meets PLOS ONE's **style requirements**, including those for file naming. The PLOS ONE style templates can be found at https://journals.plos.org/plosone/s/file?id=wjVg/PLOSOne_formatting_sample_main_body.pdf and https://journals.plos.org/plosone/s/file?id=ba62/PLOSOne_formatting_sample_title_authors_affiliations.pdf

*Authors Response:*

Manuscript has been adjusted by the journal’s requirement.

2. Your ethics statement should only appear in the Methods section of your manuscript. If your ethics statement is written in any section besides the Methods, please move it to the Methods section and delete it from any other section. Please ensure that your ethics statement is included in your manuscript, as the ethics statement entered into the online submission form will not be published alongside your manuscript.

*Authors Response:*

The ethics statement has been moved from *page 19, line 389* to *page 6 line 116-118*.

3. In the online submission form, you indicated that data cannot be shared publicly because of ethical considerations. Data are available from the corresponding author Institutional Data Access / Ethics Committee for researchers who meet the criteria for access to confidential data.

All PLOS journals now require all data underlying the findings described in their manuscript to be freely available to other researchers, either 1. In a public repository, 2. Within the manuscript itself, or 3. Uploaded as supplementary information.

This policy applies to all data except where public deposition would breach compliance with the protocol approved by your research ethics board. If your data cannot be made publicly available for ethical or legal reasons (e.g., public availability would compromise patient privacy), please explain your reasons on resubmission and your exemption request will be escalated for approval.

*Authors Response:*

We used the option Choose the declaration “data available on request” (not all data are available without restrictions) at the section “Data Availability” of the online submission form when resubmitting.

4. We note that the grant information you provided in the ‘Funding Information’ and ‘Financial Disclosure’ sections do not match. When you resubmit, please ensure that you provide the correct grant numbers for the awards you received for your study in the ‘Funding Information’ section.

*Authors response:*

The Grant Number was added at the section “Financial Disclosure” of the online submission form.

5. Please note that funding information should not appear in any section or other areas of your manuscript. We will only publish funding information present in the Funding Statement section of the online submission form. Please remove any funding-related text from the manuscript.

*Authors response:*

We removed the funding-related content listed under the subtitle “funding”, at page 20, lines 398-400

6. If the reviewer comments include a recommendation to cite specific previously published works, please review and evaluate these publications to determine whether they are relevant and should be cited. There is no requirement to cite these works unless the editor has indicated otherwise.

**Reviewer's Responses to Questions**

# Reviewer #1

**Introduction:**

1.Literature gap justification: The introduction mentions that "it is not yet clear how these factors are related to physical activity levels in pre(frail) older adults especially those living in rural areas." However, could you provide more specific evidence about what aspects of this relationship remain unexplored, given the existing literature on frailty and physical activity?

*Authors response:*

While the existing literature has established correlations between individual health factors and physical activity in the general population of older adults and frail older adults (Wang et al, 2023; Liu et al, 2024), several critical aspects of this relationship remain unexplored, particularly in our target population. Our study addresses these specific gaps:

- The examination of complex interactions between different health factors, moving beyond simple correlations to understand how these factors may moderate or mediate each other's effects on physical activity, examining how perceived health across these domains influences physical activity levels in an integrated evaluation rather than as isolated factors;
- The focus on pre-frail and frail older adults specifically living in rural areas, a population systematically underrepresented in research despite their unique barriers to physical activity and healthcare access;
- Although multiple factors have been associated with physical activity, previous studies often rely on traditional regression methods, which have seldom quantified the relative predictive importance of these factors or captured potential non-linear relationships. Our study applies machine learning (XGBoost) alongside SHAP analysis to move beyond associative insights and quantify the unique contribution of each factor within a multidimensional framework. This approach allows us to identify the most salient determinants of physical activity in this population;
- Large-scale cohort studies in China (Yanfei et al, 2023), such as the CHARLS (China Health and Retirement Longitudinal Study), have predominantly relied on questionnaire-based data, which lack objectively measured health indicators (e.g., SPPB, TUG). This limitation may reduce the explanatory power of findings related to physical function and frailty. Our study incorporates both self-reported and performance-based measures to provide a more comprehensive understanding.

To address this point, we added text and references in the introduction section *page 5, line 101 - 105*, *"While studies have addressed relationships between various individual health factors and physical activity among older adults using regression approaches, the integration of biological, psychological, and social domains and assessment of interactions among these factors will provide a more comprehensive picture of the relation between physical activity and different health domains."*

- Wang Y, Wang X, Zhu X, Sun Y, Han B, Chen T. Physical activity, physical frailty and depressive symptoms among Chinese male and female older adults: do different indicators make a difference? Front Public Health. 2023 Nov 27;11:1280952. doi: 10.3389/fpubh.2023.1280952. PMID: 38089035; PMCID: PMC10711064.
- Liu R, Menhas R, Saqib ZA. Does physical activity influence health behavior, mental health, and psychological resilience under the moderating role of quality of life? Front Psychol. 2024 Mar 11;15:1349880. doi: 10.3389/fpsyg.2024.1349880. PMID: 38529092; PMCID: PMC10961448.
- Yanfei Guo, Nawi Ng, Sven Hassler, Fan Wu, Junmei Miao Jonasson, Frailty Trajectories in Chinese Older Adults: Evidence From the China Health and Retirement Longitudinal Study, Innovation in Aging, Volume 8, Issue 1, 2024, igad131, https://doi.org/10.1093/geroni/igad131

2.Hypothesis development: The hypothesis states that "self-perceived health will have a major impact on physical activity levels compared to other health-related factors." What theoretical framework or preliminary evidence supports prioritizing self-perceived health over other established predictors like social support or physical performance?

*Authors response:*

The concept of perceived health is grounded the Biopsychosocial Model of Health as formulated by Engel et al 1977, which posits that health and illness emerge from complex, dynamic interactions among biological, psychological, and social factors rather than solely from physiological dysfunctions (Bolton et al, 2023).

While self-reported health may not accurately reflect physical capacity (Cells-Morales et al., 2021), for older and frail adults, both objective measures (mobility, comorbidities, frailty indices) and self-perceived health (self-rated health, self-efficacy, perceived fall risk) significantly influence physical activity levels. Objective factors like muscle strength, walking distance, mobility and comorbidity can enable or limit activity, while psychological and social constructs (such as self-rated health, self-efficacy, and social support) shape motivation, interpretation, and behavior (Beyer et al, 2015; Deshpande et al, 2008). Integrating both dimensions offer a holistic understanding that aligns with established health-behavior theories and evidence.

A recent study emphasizes that among community-dwelling older adults, those reporting better perceived health were more likely to maintain regular physical activity, regardless of objective health limitations (Sodergen et al, 2008). Another study conducted in frail populations, perceptions of fitness and confidence in movement (self-efficacy) influence willingness to engage in activity even when objective physical limitations are present (McAuley et al, 2006).

To clarify this point, we added in the manuscript *page 5 line 92 - 98, “As indicated by the Biopsychosocial Model of Health, both objective health measures and self-perceived health significantly influence physical activity (17). While objective factors such as grip strength and walking speed establish physical capacity, self-perceived health including self-rated health, self-efficacy, and social support, play a critical role in motivating and sustaining activity (18, 19). A growing body of evidence supports the integration of these dimensions to better understand physical activity behavior within a holistic biopsychosocial framework, especially in understudied populations (20, 21).”*

And we fine-tuned the hypothesis on *page 6, line 108 - 112* to “*Based on the biopsychosocial model of health, we hypothesize that self-perceived health measures will demonstrate associations with physical activity levels among pre-frail and frail older adults, and that the interaction between psychological, biological, and social factors will explain a significantly greater proportion of variance in physical activity than any single domain.*

- Bolton D. A revitalized biopsychosocial model: core theory, research paradigms, and clinical implications. Psychol Med. 2023 Dec;53(16):7504-7511. doi: 10.1017/S0033291723002660.
- Beyer AK, Wolff JK, Warner LM, Schüz B, Wurm S. The role of physical activity in the relationship between self-perceptions of ageing and self-rated health in older adults. Psychol Health. 2015;30(6):671-85. doi: 10.1080/08870446.2015.1014370.
- Deshpande N, Metter EJ, Lauretani F, Bandinelli S, Guralnik J, Ferrucci L. Activity restriction induced by fear of falling and objective and subjective measures of physical function: a prospective cohort study. J Am Geriatr Soc. 2008 Apr;56(4):615-20. doi: 10.1111/j.1532-5415.2007.01639.x.
- John JM, Haug V, Thiel A. Physical Activity Behavior from a Transdisciplinary Biopsychosocial Perspective: a Scoping Review. Sports Med Open. 2020 Oct 17;6(1):49. doi: 10.1186/s40798-020-00279-2.
- Komáromi L, Somogyi A, Szabo A. The Biopsychosocial Health Model Differentiates Long-Term Exercisers From Non-Exercisers: A Cross-Sectional Study. Health Sci Rep. 2025 Feb 19;8(2):e70476. doi: 10.1002/hsr2.70476..
- Zhang T, Lu G, Wu XY. Associations between physical activity, sedentary behaviour and self-rated health among the general population of children and adolescents: a systematic review and meta-analysis. BMC Public Health. 2020 Sep 3;20(1):1343. doi: 10.1186/s12889-020-09447-1.
- Södergren M, Sundquist J, Johansson SE, Sundquist K. Physical activity, exercise and self-rated health: a population-based study from Sweden. BMC Public Health. 2008 Oct 7;8:352. doi: 10.1186/1471-2458-8-352.
- McAuley E, Konopack JF, Morris KS, Motl RW, Hu L, Doerksen SE, Rosengren K. Physical activity and functional limitations in older women: influence of self-efficacy. J Gerontol B Psychol Sci Soc Sci. 2006 Sep;61(5):P270-7. doi: 10.1093/geronb/61.5.p270
- Celis-Morales CA, Perez-Bravo F, Ibañez L, Salas C, Bailey ME, Gill JM. Objective vs. self-reported physical activity and sedentary time: effects of measurement method on relationships with risk biomarkers. PLoS One. 2012;7(5):e36345. doi: 10.1371/journal.pone.0036345.

3.Geographic specificity: The introduction emphasizes rural China, but how do the unique characteristics of Northeast China (climate, culture, economic conditions) potentially influence the generalizability of findings to other rural contexts?

*Authors response:*

As indicated by the reviewer, "the rural area" doesn't exist as a single entity. For instance, the rural area of Northeast China is characterized by several unique features. Environmentally, it has a severe continental monsoon climate with long, harsh winters, extremely low temperatures, and heavy snowfall. In contrast, southern rural regions in China (e.g., the Pearl River Delta or Yunnan-Guizhou Plateau) have a much milder climate that permits year-round outdoor activities. Socially, the Northeast region has a strong collectivist mindset stemming from its state-owned enterprise history. It has distinctive local dialects, traditions, and a community-oriented atmosphere. The region has experienced economic decline and population loss, resulting in an aging rural demographic. These characteristics may affect generalizability of the results of the study, However, despite cultural and social differences between rural areas, there are some common differences in physical activity patterns between urban and rural older adults. Rural seniors engage more often in agricultural work and household chores but face challenges including limited infrastructure, greater distances, poor transportation, and isolation.

Moreover, this study does not aim to provide a one-size-fits-all conclusion, but rather to offer deep insights within a specific context. Its value comes from uncovering potential relationships and establishing a reference point for comparable research in other regions.

To acknowledge this point, we added in the discussion section a paragraph, on *page 18, line 394 – 400*: “*It is worth noting that rural environments vary considerably across regions. For instance, Northeast China's rural areas characterized by severe continental monsoon climates with extended sub-zero winters, creating distinct living conditions compared to southern regions' milder year-round climate. These geographical variations, along with distinct cultural practices and social structures across different rural communities, may influence the generalizability of our findings. Nevertheless, our findings provide a valuable methodological framework that can inform future comparative studies across diverse rural settings.*

**Methods**

4.Frailty classification consistency: The inclusion criteria require "frailty score >0 according to the Fried Phenotype," but later analysis combines pre-frail and frail participants. How might this heterogeneity within the sample affect the interpretation of physical activity predictors, and should these groups be analyzed separately?

*Authors response:*

Indeed, the inclusion criteria were a Fried score >0, meaning all included participants were pre-frail or frail according to Fried criteria. *Page 17 line 342* states: “All participants in this study were classified as physically (pre)frail according to the Fried frailty criteria, with the majority exhibiting slow gait speed (99.3%) and low grip strength (59.2%)”, This might be a confusing sentence so we changed that to “*pre-frail or frail*”. Fried factors were included in the analysis (grip strength, walking speed, fatigue, nutrition, and physical activity), which means frailty and pre-frailty were accounted for in the model without a group comparison (see also question 6 XGBoost explanation).

5. IPAQ-SF validity concerns: Given that you acknowledge in the discussion that IPAQ-SF tends to overestimate activity levels with correlations often below acceptable standards, why was this instrument chosen over more objective measures, and how might this limitation affect your primary findings?

*Authors response:*

We acknowledge the validity concerns surrounding the IPAQ-SF, as it has been shown to overestimate physical activity levels. However, as indicated in the answer to question 1, we worked from a biopsychosocial framework, which also includes self-reported measures. Ideally, these should have been combined with additional objective measures of physical activity intensity such as wearable accelerometers. Several practical and methodological considerations specific to our study context in rural Northeast China made this not possible.

- The adherence to wearable devices is notably low in this population, as reported in a recent study (Ming et al., 2025). This implies that additional efforts and implementation strategies are required to increase adherence, which were beyond the scope of the present study.
- Resource limitations and the logistical challenges of conducting research in rural settings restricted the feasibility of distributing and maintaining wearable devices. Even if devices were distributed, their successful long-term use would be challenging. Short wear time and frequent removal would significantly compromise data quality and continuity (often caused by discomfort with wearing the device, particularly during sleep, or the necessity to remove it in moist environments followed by failure to put it back on). In contrast, the IPAQ-SF, though subject to recall bias, provides a standardized and feasible method to capture typical weekly activity patterns across a large sample.
- Financial and logistical constraints cannot be overlooked. Providing robust, user-friendly devices for each participant and ensuring continuous monitoring and technical support would be prohibitively expensive and operationally complex within the constraints of this study.

Therefore, IPAQ-SF was chosen as a pragmatic solution given its wide use, international comparability, and feasibility for large-scale population-based studies. To mitigate recall bias and potential overreporting, we employed a one-on-one short interview format in which investigators explained the exact meaning of each question before recording participants’ responses. This approach minimized misunderstandings and enhanced the accuracy of self-reported data.

We have also addressed at the Method section on *page 6 line 127 - 130* “*Considering the low education level of most participants, all questionnaires and self-reported measurements were conducted in a one-on-one question-and-answer format and recorded by the investigators.”*

Nonetheless, we acknowledge that these methodological limitations may affect the interpretation of our findings, particularly concerning the absolute levels of physical activity. Accordingly, we have explicitly addressed this issue in the Discussion section, on *page 19, line 418 -422* “*The results reflect self-reported rather than objectively measured physical activity. While the associations identified between variables remain meaningful within the framework of this study, we recommend that future research incorporate device-based measures to further validate and extend these findings (46).*”

Ming Y, Li Y, Liu Y. Digital technologies as solutions to China's aging population: a systematic review of their opportunities and challenges in rural development. Front Public Health. 2025 Jan 15;12:1416968. doi: 10.3389/fpubh.2024.1416968.

6.XGBoost model justification: While you mention XGBoost can handle imbalanced data and non-linear relationships, what specific advantages does this approach offer over traditional logistic regression for this particular research question, especially given the relatively small sample size (n=284)?

*Authors response:*

XGBoost excels in handling high-dimensional data relative to sample size. While logistic regression can suffer from the "curse of dimensionality" and may overfit with too many features, XGBoost has built-in regularization parameters that help prevent overfitting.

- Parameters like max_depth, min_child_weight, and gamma control tree complexity
- The learning rate parameter reduces individual trees' influence, improving generalization
- The boosting algorithm iteratively improves the model by focusing on previously misclassified samples
- XGBoost achieves higher accuracy with fewer samples compared to single models, and early stopping prevents overfitting by monitoring validation performance

XGBoost also offers feature importance scoring to identify which variables contribute most to predictions and works effectively with sparse matrices for high-dimensional data. Unlike logistic regression—a linear model assuming a linear relationship between variables and outcome log-odds—XGBoost is a tree-based ensemble method that captures complex non-linear patterns and interaction effects between variables without explicitly specifying them. With small datasets, XGBoost remains relatively robust to outliers due to its tree-based architecture, while logistic regression is more sensitive to extreme values.

Despite its advantages, XGBoost has limitations: more time-consuming hyperparameter tuning and less interpretability compared to logistic regression.

We've briefly outlined this on *page 9 line 196* and added three references. and chose not to elaborate further as it would place too much emphasis on the methodology. Two of the cited studies (Yan et al., 2022, Wang et al., 2022) reports that XGBoost demonstrates superior prediction performance compared to logistic regression, while the other one (Athanasiou et al, 2020) indicates that XGBoost is effective in handling both imbalanced and non-linear data.

- Yan Z, Wang J, Dong Q, Zhu L, Lin W, Jiang X. XGBoost algorithm and logistic regression to predict the postoperative 5-year outcome in patients with glioma. Ann Transl Med. 2022 Aug;10(16):860. doi: 10.21037/atm-22-3384..
- Wang R, Wang L, Zhang J, He M, Xu J. XGBoost Machine Learning Algorism Performed Better Than Regression Models in Predicting Mortality of Moderate-to-Severe Traumatic Brain Injury. World Neurosurgery. 2022 Jul;163:e617-e622. DOI: 10.1016/j.wneu.2022.04.044.
- M. Athanasiou, K. Sfrintzeri, K. Zarkogianni, A. C. Thanopoulou and K. S. Nikita, "An explainable XGBoost–based approach towards assessing the risk of cardiovascular disease in patients with Type 2 Diabetes Mellitus," 2020 IEEE 20th International Conference on Bioinformatics and Bioengineering (BIBE), Cincinnati, OH, USA, 2020, pp. 859-864, doi: 10.1109/BIBE50027.2020.00146.

**Results**

7.Class imbalance impact: Despite using class weighting, your model shows 90% specificity but only 63% sensitivity. How does this imbalance affect the practical· utility of the model, particularly for identifying individuals who might benefit from physical activity interventions?

*Authors response:*

The class imbalance in our dataset reflects the (true) prevalence of physical inactivity in the general rural population. Our further use of weighted analysis was a technical step to ensure the model learned from the minority class and did not simply default to predicting the majority class. However, we recognize that specificity (90%) and sensitivity (63%) constrain the model’s ability to detect all individuals at risk of low physical activity. While the high specificity supports efficient resource allocation, the lower sensitivity means that some individuals who could benefit from interventions may be overlooked. In an ideal world with unlimited resources, we would strive to identify every single eligible individual. However, in real-world community or clinical settings, resources (e.g., health worker time, program slots) are severely constrained. The key practical implication of our model's high specificity (90%) is that it creates a high-priority subgroup where 9 out of 10 predicted individuals are truly inactive. This allows decision-makers to allocate their limited interventions with high efficiency and confidence, knowing that resources are not being wasted on false alarms.

- The Cost of a False Positive (Prioritized by our model): Wasting a precious intervention slot on an already active individual. This is a direct drain on limited resources and reduces the overall cost-effectiveness of the public health program.
- The Cost of a False Negative (Acknowledged by our model): An inactive individual is not identified in this first round. However, given that physical activity promotion is an ongoing public health effort, these individuals may be identified through other routes (e.g., routine primary care visits, broader community campaigns) or in subsequent screening cycles. The consequence is a delay in intervention, not a permanent loss of opportunity.

Therefore, for the specific setting of current study, we think that minimizing false positives (high specificity) is more pragmatically critical than minimizing false negatives (high sensitivity).

In future work, this model could serve as the first, highly specific filter. To enhance the model’s ability to capture more at-risk individuals, those identified as “high activity” could then undergo a second, less resource-intensive screening (e.g., a single question from a GP) to catch potential false negatives.

To address this point clearly in the discussion, the following content has been added on *page 17 line 368 - 373 “Therefore, this model is positioned as a screening tool, rather than diagnostic, enabling healthcare providers to efficiently identify a high-risk subgroup for physical inactivity and prioritize limited resources toward those most in need. Future research should prioritize improving sensitivity, through methodological refinements, incorporating additional behavioral and environmental variables, to maintain acceptable specificity*.”

8.Feature importance interpretation: The SHAP analysis shows SSRS (social support) as highly influential, but Figure 4 shows considerable overlap between groups. How do you explain this apparent contradiction, and what does this suggest about the model's ability to capture the true relationship?

*Authors response:*

The apparent contradiction between the high overall importance of the SSRS and the overlap in its SHAP value distribution is not an error but rather a key characteristic of its relationship with PA in our study.

The SHAP summary plot ranks features based on their average impact on the model's output magnitude across all individuals. A high SHAP value for SSRS indicates that, on average, variations in social support scores cause large shifts in the model's prediction across the entire dataset. This confirms that SSRS is a powerful predictor overall.

The overlap in Figure 4 illustrates the heterogeneity and non-linear nature of this relationship. It shows that the same level of social support can have different effects on different individuals. For example: some individuals with moderate SSRS scores, other factors (e.g., poor perceived health or low physical performance) may dominate, resulting in a low predicted PA level (negative SHAP value); others with similar SSRS scores, positive factors (e.g., good mental health or strong functional capacity) may interact with social support, leading to a high predicted PA level (positive SHAP value). This overlap is a common and expected finding for influential yet non-deterministic psychosocial factors.

This pattern does not indicate a weakness of the model, instead, it reveals a complex**,** context-dependent relationship that the XGBoost algorithm is successfully capturing. The overlap suggests that social support alone is rarely sufficient to guarantee high PA. Its effect is moderated by other variables in the model. The model's strength is its ability to capture these complex interactions. The results also reflect the real-world complexity of health behaviors. It suggests that interventions targeting social support may be highly effective to PA, but their success will likely depend on simultaneously addressing other individual-specific barriers, such as functional limitations or mental health.

To address this point clearly, we also added in the discussion section on *page 16, line 338 - 341* “*The overlap between groups further indicates that social support alone is rarely sufficient to ensure high levels of physical activity. Success of interventions targeting social support depends on simultaneously addressing other individual specific barriers, such as functional limitations or perceived health status*.”

9. Physical performance paradox: Your results show that even the high physical activity group performed below the 5th percentile for age-matched peers on physical tests. How do you reconcile high self-reported physical activity levels with poor objective physical performance measures?

*Authors response:*

The paradox might be from the use of IPAQ-SF. The IPAQ-SF assesses total physical activity without distinguishing between leisure-time physical activity (LTPA) and occupational physical activity (OPA). This distinction is critical because growing evidence suggests that LTPA and OPA may have divergent effects on health. LTPA is consistently associated with improved physical function, cardiovascular health, and reduced morbidity. In contrast, prolonged, high-volume OPA, characterized by repetitive movements, heavy lifting, and inadequate recovery time, has been linked to physical weakness, musculoskeletal disorders, and chronic fatigue, potentially accelerating functional decline rather than mitigating it (Suutari-Jääskö et al, 2023).

Therefore, the "high PA-poor performance" paradox may be explained by the fact that the participants' high activity volumes are largely attributable to physically demanding and often stressful occupational work. In our study, 86% of participants were farmers, and the data collection period immediately followed the autumn harvest season, a time of intense agricultural labor. Consequently, the reported "high" activity levels predominantly reflect strenuous, prolonged occupational work (e.g., planting, lifting, harvesting) rather than voluntary, health-enhancing leisure-time exercise. This interpretation is consistent with the "physical activity health paradox" recently discussed in the literature.

We also emphasized this point in the discussion section of our revised manuscript. On *page 17 line 353 - 357*, we state: “*The IPAQ-SF assesses total physical activity without distinguishing between leisure-time physical activity (LTPA) and occupational physical activity (OPA), which may partly explain the observed paradox of high self-reported PA alongside poor physical performance. These findings addressed the necessity of considering the distinct roles of LTPA and OPA separately in future research, particularly in agricultural populations where occupational demands dominate daily activity patterns*.”.

- Suutari-Jääskö A, Parkkila K, Perkiömäki J, Huikuri H, Kesäniemi YA, Ukkola OH. Leisure time and occupational physical activity, overall and cardiovascular mortality: a 24-year follow-up in the OPERA study. Ann Med. 2023;55(2):2245429. doi: 10.1080/07853890.2023.2245429.

**Discussion**

10. Causality concerns: The discussion frequently implies causal relationships (e.g., "self-reported health, social support, and physical activity engagement"), but this is a cross-sectional study. How do you address the potential for reverse causality, where poor physical function might lead to negative health perceptions rather than vice versa?

*Authors response:*

Indeed, causality cannot be proven, because of the design of the cross-sectional. What we aim to emphasize is the correlation between physical activity (PA) and other frailty-related factors, as noted in the discussion: “However, this represents an association rather than causation.” Any confusion may have arisen from the use of causal language such as “affect” (page 2, line 33), “impact” (page 3, line 55), and “influence” (page 5, line 106; page 16, line 315; page 20, line 425). These terms were replaced with more appropriate alternatives, such as “association,” “interaction,” or “correlation”, to more accurately reflect the observational nature of the analyses.

11.Intervention implications: You suggest "community-based social support programs and structured mobility-enhancing exercises," but given that most participants already met high physical activity levels (despite poor physical performance), what specific interventions would be most appropriate for this population?

*Authors response:*

Our suggestions are indeed predicated on the key distinction, raised in our response to Comment 9, between occupational and leisure-time physical activity (PA). Given that our explanation that high levels of occupational PA are associated with poor physical performance, the primary goal of intervention is not to increase total PA volume, but to counteract the negative effects of occupational PA and promote health-enhancing movement patterns. The following strategies could be attempted:

- Health education: educating farmers on safe lifting techniques, proper postures during prolonged labor, and the importance of incorporating micro-breaks and stretching into their long working hours. The aim is to reduce the risk of musculoskeletal injury and chronic pain, which is likely to contribute to their poor performance on tests like grip strength and the Timed Up & Go.
- Building on the key finding from our SHAP analysis, which identified social support (as measured by the SSRS) as a highly influential factor, we recommend that PA programs be intentionally designed around social participation. Encouraging necessary framing activities done with neighbors, family, or other partners could help mitigate the negative health effects associated with occupational PA (OPA) by combining movement with meaningful social interaction.
- Furthermore, during less busy agricultural seasons, community-based PA programs should be promoted. These initiatives must be specifically tailored to counteract the physical imbalances and strain resulting from repetitive labor. Such programs should be framed as opportunities for social connection, relaxation, and proactive health maintenance, rather than as an extension of work.

To address the future implication, we added in the discussion section on *page 19, line 407 -412* “*For the PA intervention in this population, the primary objective is not to increase total PA volume, but to mitigate the detrimental effects of work-related movement and promote health-enhancing behaviors. Proposed strategies include health education to improve biomechanics and reduce injury risk, enhancing social participation during repetitive labor, and tailored community physical activity program during off-peak seasons designed to counteract physical strain and foster social engagement.*”

12.Methodological limitations: While you acknowledge IPAQ-SF limitations in the discussion, this appears to be a fundamental flaw that may compromise the study's validity. How significantly might measurement error affect your conclusions, and should this limitation be addressed more prominently earlier in the manuscript?

*Authors response:*

We acknowledge the limitation of the IPAQ-SF as a self-report instrument (see also answer to question 9 ) . As noted in our response to Comment 5, the IPAQ-SF was a chosen, based on several methodological and practical considerations specific to our study context in rural Northeast China. Despite its limitations, the impact on our main conclusions was minimal due to the robust mitigating measures we incorporated into our study design.

To directly address potential recall bias associated with self-reported tools like the IPAQ-SF, our study did not employ a self-administered questionnaire. Instead, all IPAQ-SF data were collected through trained interviewers conducting one-on-one interviews. This method significantly improves data quality by allowing interviewers to clarify questions, probe for accurate recall, and ensure consistent understanding across all participants, especially crucial in an older rural population.

As stated on *page 6 line 127 - 130* *“Considering the low education level of most participants, all questionnaires and self-reported measurements were conducted in a one-on-one question-and-answer format and recorded by the investigators who were trained to interview participant.”*

Nevertheless, it is important to explicitly mention the limitations of the IPAQ-SF. We addressed this in the discussion on *page 19 line 413 - 422*: "A limitation of this study pertains to the reliance on self-reported questionnaires for assessing physical activity, particularly the IPAQ-SF. This instrument was chosen because of several methodological and practical considerations specific to our study context in rural Northeast China. Although the IPAQ-SF is widely used to assess physical activity in the aging population, a recent systematic review reported that compared to objective physical activity measures with wearable sensors, the IPAQ-SF tends to overestimate activity levels (21). Therefore future studies should address how to incorporate wearable activity sensors to obtain additional objective measurements of physical activity levels across different environments and populations.

# Reviewer #2:

This manuscript describes the study of factors contributing to differences in physical activity levels in prefrail older adults in rural areas. This is an important topic since frailty prevention in older adults should be a priority in all populations.

General comments:

1. Authors should follow financial disclosure guidelines.

*Authors response:*

*The revised manuscript has been adjusted as follows:*

a) Remove the funding-related content listed under the subtitle “funding”, at page 20, line 398-400
b) Add Grant Number at the section “Financial Disclosure” of the online submission form when resubmit.

2. Language revision is required.

*Authors response:*

The complete manuscript was reviewed by a native English speaker familiar with the topic, for a comprehensive language and subject-matter.

3. Authors declared all data are available without restrictions, yet they later stated that data cannot be shared publicly. Non-identifiable data can be provided without restrictions, and the statements need to be congruent.

*Authors response:*

We used the option: “data available on request” (not all data are available without restrictions) at the section “Data Availability” of the online submission form when resubmit.

Abstract:

4. Abstract sections do not usually include a discussion, instead; they include a conclusion. Please follow Plos One guidelines.

*Authors response:*

*Page 2 Line 52, we’ve* replaced the term “Discussion” by “Conclusion”.

**Introduction:**

5. I am not sure how the Chinese government classifies age groups. Is the definition of older adults starts at the age 60 or 65? The authors included patients who were 60 or above. Yet, in the introduction, the classification states 65 years or above. Please explain why you chose to use the age 60 or more instead of official classification.

*Authors response:*

Before May of 2025, the term “older adults” means “adults who have reached the age of 60” in China, defined in chapter 1 of <**Law of the People's Republic of China on the Protection of the Rights and Interests of Older Adults**>. Because our study was conducted and completed before May of 2025, we recruited the population older than 60, not 65 years old and above.

6. Aim: Authors intended to measure the influence of different health domains on physical activity levels. Since this was a cross-sectional study, causality cannot be proven. All we can confirm here is the presence of an association. I would assume that higher physical activity can positively influence other health domains. This is more logically proven via studies on the positive influences of higher physical activity on many health-related aspects and on diseases.

*Authors response:*

Indeed, causality cannot be proven, because of the cross-sectional design of our study. What we aim to emphasise is the correlation between physical activity (PA) and other frailty-related factors, as noted in the discussion on page 16 line 311: “However, this represents an association rather than causation.” Any confusion may have arisen from the use of causal language such as “affect” (page 2, line 33), “impact” (page 3, line 55), and “influence” (page 5, line 106; page 16, line 315; page 20, line 425). These terms were replaced with more appropriate alternatives, such as “association,” “interaction,” or “correlation”, to more accurately reflect the observational nature of the analyses.

A key motivation for this study went beyond establishing correlations. We compared two groups with (pre)frailty status (according to Fried criteria) that significantly differed in physical activity levels. Our goal was to identify factors—particularly psychosocial and behavioral characteristics—that distinguish these groups and might explain why some frail older adults maintain higher activity levels. This approach helps identify potential modifiable factors (such as social support, self-efficacy, or specific health perceptions) that future interventions could target, even when frailty is present.

As stated in the study objective on *page 5, line 106 - 108*: *"This study aims to gain insight into the influence of different health domains on physical activity levels in older adults living in rural areas of China who have been identified as physically pre-frail or frail."*

**Methods:**

7. Including all those with Fried’s criteria scores more than 0 means including patients with frailty as well.

*Authors response:*

Indeed, we included older adults who were classified as pre-frail or frail according to the Fried frailty phenotype. While the Fried scale provides a useful measure, frailty is inherently a multidimensional concept that exists along a continuum. Therefore, to capture this full spectrum, we included participants who were both pre-frail and frail (see also our response to Reviewer 1, Comment 4).

8. Please provide the approval to use MMSE as it is copy righted.

*Authors response:*

We have not included any items or the full text of the MMSE in our manuscript; only the scores or outcomes of the administered tests have been used. The Mini-Mental State Examination (MMSE) is widely used in clinical and research settings and was administered in our study in the usual context. In the text of the manuscript, we have explicitly referenced the original source and the copyright holder: Folstein et al., 1975, including the publisher.

Although freely accessible cognitive screening instruments could be considered for future studies, it was necessary to use the MMSE in this dataset because it had already been administered to the participants.

Folstein MF, Folstein SE, McHugh PR. "Mini-mental state". A practical method for grading the cognitive state of patients for the clinician. J Psychiatr Res. 1975 Nov;12(3):189-98. doi: 10.1016/0022-3956(75)90026-6. PMID: 1202204.

9. Authors included anxiety, depression and social support under perceived health item in the results. Psychiatric problems are not the only contributors to health and not including physical problems as well can gravely affect the results. Please include other aspects of health in your analysis.

*Authors response:*

Indeed, psychiatric conditions are not the sole determinants of health, factors from other domains can also significantly influence health status. Therefore, we included variables across four domains, as stated on *page 7 lines 153 - 181*:

1) Demographic and personal characteristics: age, waist-hip ratio (WHR), body mass index (BMI), gender, occupation, education level, income, marital status, and living arrangement;
2) Health behavior variables: smoking, alcohol consumption, daily fiber intake, and daily protein intake;
3) Objective health measures: grip strength, walking speed assessed via a 3-meter walking test, Timed Up and Go test (TUG) duration, total score on the Short Physical Performance Battery (SPPB), and number of comorbidities based on health records;
4) Self-reported health perception variables: scores from the Generalized Anxiety Disorder-7 (GAD-7) (24), Patient Health Questionnaire-9 (PHQ-9) (25), and Social Support Rating Scale (SSRS).

Physical health-related factors were categorized under objective health measures. In total, 26 factors were assessed and collected in this study. It was not feasible to include additional factors beyond these 26 factors. The analysis must rely solely on the existing dataset, more other aspects health factors are not available in the already collected dataset.

**Results:**

10. Where are the supporting tables? I was not able to find them.

*Authors response:*

The supporting information appears to have been unintentionally omitted from the final submission file. The Supplementary Material can be found at the end of the rebuttal, and we will promptly resubmit the complete supporting information as well.

11. In table 1: authors did not put the significance of all items. Only significant variables had their p values in the table. Please add all the data in the table.

*Authors response:*

We’ve added statistical metrics for all of the factors. The revised Table 1 is as follows:

| **Table 1: Demographics and outcome variables of the two groups** | | | | |
| --- | --- | --- | --- | --- |
| Variables (unit) | Low-Moderate PA | High PA | Statistic | |
| N | 84 | 200 | - | - |
| Age (y) | 70.5 (5.6) | 69.2 (5.4) | U = 1.07 | *p* = .380 |
| Gender Female vs Male | 58% | 60% | Fisher | *p* = .960 |
| Occupation Farmer vs. other | 74% | 91% | *Χ²* =10.65 | *p* = .001 |
| Education < 6 year | 79% | 79% | Fisher | *p* = 1.000 |
| Income < 3000 (￥/m) | 90% | 96% | *Χ²* = 4.29 | *p* = .038 |
| Married vs other | 76% | 81% | Fisher | *p* = .449 |
| Living with others Yes | 85% | 90% | Fisher | *p* = .328 |
| Smoking No | 45% | 33% | *Χ²* = 4.15 | *p* = .042 |
| Drinking No | 77% | 79% | Fisher | *p* = .959 |
| Daily Fiber Yes | 82% | 87% | Fisher | *p* = .447 |
| Daily protein Yes | 43% | 52% | Fisher | *p* = .230 |
| Comorbidities <=5 | 82% | 92% | *Χ²* = 5.91 | *p* = .015 |
| Walk >500 m | 58% | 85% | *Χ²* = 22.70 | *p* < .001 |
| Can walk stairs (functional strength) | 57% | 79% | *Χ²* = 14.19 | *p* < .001 |
| Good general health | 35% | 65% | *Χ²* = 21.54 | *p* < .001 |
| No cognitive impairment | 54% | 69% | Χ² = 5.73 | *p* = .017 |
| WHR | 0.92 (0.06) | 0.94 (0.85) | U = 1.01 | *p* = .314 |
| BMI | 23.40 (4.00) | 24.65 (3.50) | U = 2.19 | *p* = .028 |
| GP (kg) | 19.64 (8.48) | 21.99 (8.51) | U = 1.87 | *p* = .030 |
| WS (m/s) | 0.60 (0.21) | 0.67 (0.21) | U = 3.28 | *p* = .001 |
| TUG (seconds) | 14.98 (7.91) | 12.06 (3.25) | U = 2.43 | *p* = .020 |
| SPPB - | 6.67 (2.66) | 8.20 (2.34) | U = 4.40 | *p* < .001 |
| MMSE | 19.67 (4.96) | 21.81 (4.90) | U = 3.16 | *p* = .002 |
| PHQ9 | 8.31 (5.43) | 6.67 (4.57) | U = 2.31 | *p* = .021 |
| GAD7 | 7.31 (5.70) | 6.88 (5.35) | U = 0.61 | *p* = .541 |
| SSRS | 32.18 (7.71) | 36.35 (7.39) | U = 4.12 | *p* < .001 |
| PA = Physical Activity; WHR = Waist-Hip Ratio; BMI = Body Mass Index; GP = Grip strength; WS = Walking Speed; TUG = Timed-up-and-Go; SPPB = Short Physical Performance Battery; MMSE = Minimal Mental State Examination; PHQ9 = Patient Health Questionnaire; GAD7 = General Anxiety Disorder Questionnaire Score; SSRS = Social Support Rate Scale; Χ² = Chi-square test; U = Mann Withney U test. | | | | |

**Discussion:**

12. Please cite the authors by name when it is part of the sentence structure (Page 25 line 324).

*Authors response:*

Maybe the reviewer means the sentence placed at *page 16 line 346*, which is “However, normative data provided by (30) indicate that even participants ……”, this sentence has been improved to “However, normative data from Mayhew et al. indicate that even participants…… (30)”. This remark triggered us to look at omissions of the authors names in similar sentence structures. In this way we found two similar issues and addressed this accordingly (see page 15, line 324 and page 19 line 369).

**Supplementary Material - Appendixes**

**Table S1**: Measurement Instruments and variables

| Variables (unit) | Measurement | Meaning |
| --- | --- | --- |
| Age | records | >= 60 years old |
| Sex | records | 1=female; 0=male |
| Occupation | records | 1=Farmer; 0=Worker/Individual Business/Driver/Retirement/Unemployed |
| Education (year) | records | 1= <=6y; 0 = >6y |
| Income (￥/m) | records | 1=<3000; 0=>=3000 |
| Marriage | records | 1=Married; 0=Single/Divorced;/Widow |
| Living | records | 1=Alone; 0=Cohabitation/With Children/Others |
| Smoking | self-reported | 1=yes; 0=no |
| Drinking | self-reported | 1=yes; 0=no |
| Daily fiber | self-reported | 1=yes; 0=no |
| Daily protein | self-reported | 1=yes; 0=no |
| Comorbidity | self-reported | 1=>=5 disease; 0=<5 diseases |
| Ambulation | self-reported | 1=could walk 500 meter;  0= could not walk 500 meter |
| Functional strength | self-reported | Could go up a one floor stair or a 10-meter slope  1= yes  0=no |
| General Health | self-reported | 1=Bad;  0=Well/Good/Great/Excellent |
| Cognitive impairment (CI) | physical test | 0=no; 1=yes; defined by total score of MMSE and education level; <18 for illiterate; <21 for 1-6 years of education; <25 for 7 or more years of education |
| WHR | physical test | Calculated by waistline (cm)/hipline (cm) |
| BMI | physical test | Calculated by weight/(height/100)2 |
| GP (kg) | physical test | Maximum value of two measurements in each hand |
| WS (m/s) | physical test | Walking speed, measured by 3m walking test |
| TUG (seconds) | physical test | Time of Time Up and Go test – total time |
| SPPB - | physical test | Total score of the Short Physical Performance Battery. Range 0-12  12 points: Highest performance, indicating optima lower extremity function and mobility.  0-3 points: Very low performance, indicating severe functional impairments and a high likelihood of disability, falls, or dependency. |
| MMSE | cognitive test | Total score of mini-mental state examination, range 0-30  See CI |
| PHQ-9 | self-reported | Total score of Patient Health Questionnaire-9, range 0-27 –  0–4: Minimal or no depression;  5–9: Mild depression;  10–14: Moderate depression;  15–19: Moderately severe depression;  20–27: Severe depression |
| GAD-7 | self-reported | Total score of General Anxiety Disorder Questionnaire-7, range 0-21;  0–4: Minimal anxiety  5–9: Mild anxiety  10–14: Moderate anxiety  15–21: Severe anxiety |
| SSRS | self-reported | Total score of Social Support Rate Scale questionnaire score, range 0-66 - The total score for each domain can range from 0 to 100,  Low Score – Indicates weak social support and possible isolation.  Moderate Score – Suggests a moderate level of perceived social support.  High Score – Reflects strong social support, which is beneficial for emotional and psychological well-being. |
| IPAQ-SF | self-reported | Physical activity level calculated by Short Form International Physical Activity  1=low;  2=moderate;  3=high. |

**Table S2**: Hyperparameters settings for the XGBoost model

| Parameter | Tuning range | Best parameter |
| --- | --- | --- |
| Eta | 0.005, 0.01, 0.05, 0.1 | 0.01 |
| Max depth | 2, 4, 6, 8 | 8 |
| Nrounds | 50,100, 200 | 200 |
| Gamma | 0.005, 0.1, 1, 3 | 0.005 |
| Colsample_bytree | 0.3, 0.5, 0.7, 0.9 | 0.7 |
| Min child weight | 1, 3, 5, 7 | 1 |
